# BAPoma, a rare nevus, as the key to a diagnosis of *BAP1*‐associated tumor predisposition syndrome

**DOI:** 10.1111/ddg.15791

**Published:** 2025-05-27

**Authors:** Lara Racz, Sandra Pasternack‐Ziach, Isabel Spier, Stephan Forchhammer, Claudia Rehkämper, Peter Kind, Julia Reifenberger, Silke Redler

**Affiliations:** ^1^ Institute of Human Genetics Medical Faculty and University Hospital Düsseldorf Heinrich‐Heine‐University Düsseldorf Düsseldorf Germany; ^2^ Institute of Human Genetics University of Bonn Medical Faculty and University Hospital Bonn Bonn Germany; ^3^ National Center for Hereditary Tumor Syndromes University Hospital Bonn Bonn Germany; ^4^ Department of Dermatology University of Tübingen Tübingen Germany; ^5^ Laboratory for Dermatohistology Offenbach am Main Germany; ^6^ Department of Dermatology Medical Faculty and University Hospital Düsseldorf Heinrich‐Heine‐ University Düsseldorf Düsseldorf Germany

Dear Editors,

A 23‐year‐old female presented to our clinic with progressively changing nevi. Skin cancer screening, including body mapping, was performed, and three suspicious nevi were excised from both the dorsal and ventral flanks. The first two were found to be junctional nevi. However, the histology of the tissue from the ventral flank revealed a melanocytic nevus with partial *BAP1*‐inactivation (BAPoma) (Figure 1). Immunohistochemistry of the latter revealed a loss of nuclear *BAP1* expression (Figure 1). DNA was extracted from peripheral blood leucocytes. Sequencing and deletion/duplication screening of BRCA1‐Associated Protein 1 (*BAP1*) was performed in accordance with German gene diagnostic law. This revealed the heterozygous pathogenic germline variant c.1813G>T;p.(Glu605Ter), which results in a premature stop codon. A diagnosis of *BAP1*‐associated tumor predisposition syndrome (BAP1‐TPS) was made. In the present index patient, assessment of the family history over three generations revealed a familial clustering of tumors, some of which manifested in early adulthood (Figure 2). Interestingly, at the age of 23 years, the paternal cousin (III:3) had undergone excision of two suspicious skin lesions, which were found to be BAPomas. The father (II:1) had developed urothelial carcinoma of the urinary bladder at the age of 47 years, followed by metastatic recurrence. At the age of 54 years, a diagnosis of pleural mesothelioma was made. In addition, the patient's father had undergone excisions of numerous basal cell carcinomas since then. The paternal aunt (II:2) had 50 to 60 colonic adenomatous polyps removed since the age of 47 years, but carried no pathogenic germline variant in known polyposis‐associated susceptibility genes. She had at least three basal cell carcinomas excised from the neck area between the ages of 40 and 45 years. The paternal grandfather had died from renal cell carcinoma at the age of 75 years, and his sister had died from metastatic breast cancer at the age of 41 years. Thus, segregation of the familial *BAP1* variant was no longer possible. Except for a paternal cousin (III:4), all tested family members carried the familial *BAP1* variant (Figure 2).

**FIGURE 1:**
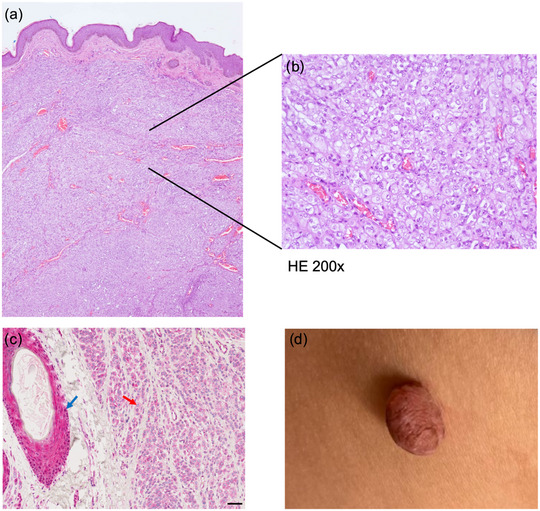
Histology, immunohistochemical staining for BRCA1‐associated protein‐1 (BAP1), clinical picture of the BAPom. (a) Beneath an inconspicuous epidermis, numerous tumor tissue with a nodular growth pattern is present in the corium, as permeated by numerous blood vessels. (b) Higher magnification reveals nests and strands of relatively monomorphic epithelioid cells with isolated mitoses (top right). This histology is compatible with partial BAPI inactivation (hematoxylin‐eosin stain, original magnification x 200). (c) Nevus components show a loss of nuclear expression for BAP1 (red arrow). Internal control tissue shows the preserved nuclear expression in the area of the hair follicle epithelium (blue arrow) (scale bar: 50 µm). (d) Pedunculated tumor on the ventral flank, approximately 0.7 cm in diameter, with irregular brownish pigmentation.

**FIGURE 2:**
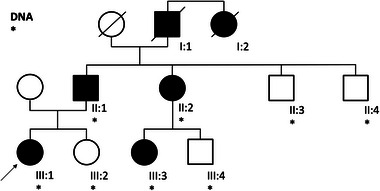
Family Pedigree. Individuals affected by malignant or benign tumors are depicted with black circles (female) and squares (male). A line through the symbol indicates that the respective individual is deceased. Asterisks indicate individuals for whom DNA was available. The index patient (III:1) is marked with an arrow. With the exception of a paternal cousin (III:4), all tested individuals carried the *BAP1* variant c.1813G>T;p.(Glu605Ter). Paternal grandfather (I:1): renal cell carcinoma, died at the age of 75 years; sister of the paternal grandfather (I:2): metastatic breast cancer, died at the age of age 41 years; father (II:1): urothelial carcinoma of the urinary bladder, diagnosed at the age of 47 years, followed 4 years later by a metastatic recurrence. Pleura mesothelioma, diagnosed at the age of 54 years. In addition, he had since undergone excision of numerous basal cell carcinomas; paternal aunt (II:2): excision of 50 to 60 adenomatous colonic polyps since the age of 47 years, removal of at least three basal cell carcinomas from the neck area between the ages of 40 and 45 years; paternal uncle (II:3): unaffected at the age of 56 years; paternal uncle (II:4): unaffected at the age of 58 years; index (III:1): melanocytic nevus with partial BAP1‐inactivation (BAPoma) on the right flank at the age of 22 years; sister (III:2): unaffected at the age of 19 years; paternal cousin (III:3): two BAPomas (abdomen, neck) at the age of 24 years; paternal cousin (III:4): unaffected at the age of 28 years.

*BAP1*‐associated tumor predisposition syndrome was first described in 2011.^1^ This rare familial cancer syndrome shows autosomal dominant inheritance, and involves pathogenic germline variants in *BAP1*, whose main function is tumor suppression at molecular levels. *BAP1* was named based on its interaction with *BRCA1* (BReast CAncer Gene 1), which ensures the repair of double‐stranded DNA breaks and genomic stability.^2^ *BAP1* is recruited to double‐strand DNA break sites and promotes repair by homologous recombination by facilitating BRCA1 recruitment.^3^ Moreover, it is part of the polycomb group repressive deubiquitinase complex (PR‐DUB), which removes ubiquitin from histones (H2AK119ub), regulating gene transcription. Loss of *BAP1* disrupts this process, resulting in altered expression of genes involved in cell cycle control, DNA damage repair, and metabolism.^4^ BAP1‐TPS is associated with predisposition to multiple tumors, including benign melanocytic cutaneous tumors and numerous malignant tumors in distinct organ systems.^5^ As with many rare TPS, it remains unclear which cancers are unequivocally associated with BAP1‐TPS. Consensus exists that the core phenotype includes BAPoma, as well as cutaneous melanoma, uveal melanoma, basal cell carcinoma, malignant mesothelioma of the pleura and peritoneum, and renal cell carcinoma. Furthermore, research has identified possible BAP1‐TPS associations with meningioma, cholangiocarcinoma, as well as breast, urinary bladder, and lung cancer.^1^, ^6^, ^7^ BAPoma is often the initial manifestation of this rare TPS.

Clinically, *BAP1*‐inactivated nevi appear as dome‐shaped, skin‐colored or reddish papules. Histologically, they typically present as dermal‐based melanocytic proliferations that are combined with prominent epithelioid cells. The latter may show nuclear pleomorphism and abundant amphophilic cytoplasm. These show an immunohistochemical loss of nuclear BAP1 expression.^8^, ^9^ Dermatologists are ideally positioned to recognize specific clinical features and to initiate germline testing and genetic counseling. This plays a crucial role in identifying high‐risk individuals for early screening strategies aimed at detecting malignant tumors in their initial stages and intercepting cancers before they become aggressive. In 2023, detailed recommendations on BAP1‐TPS clinical management were published by a panel of experts from Europe, highlighting the need for regular skin and ocular screening as well as renal imaging.^10^ The tumor spectrum described in the present report expands the phenotypic variability of BAP1‐TPS. In particular, this is the first BAP1‐TPS report to describe colonic adenomatous polyps (II:2), although we cannot exclude the possibility that the colonic adenomatous polyps developed independently of the familial *BAP1* variant.

The present case demonstrates the difficulties involved in characterizing rare tumor syndromes for which limited scientific evidence is available. Characterizing the associated tumor spectrum, establishing respective genotype‐phenotype correlations, and exploring the utility of surveillance and clinical management are crucial steps forward. In conclusion, our report describes a BAP1‐TPS family with cutaneous and non‐cutaneous tumor manifestations. BAP1‐TPS is rare, and due to its varied phenotype – even within families – BAPoma may represent a valuable tool for identification. Early recognition of BAP1‐TPS facilitates the initiation of cancer surveillance to prevent advanced malignancies.

## CONFLICT OF INTEREST STATEMENT

None.
